# The Presence of Diabetes Mellitus or Pre-diabetes Mellitus Increases Mortality from Heart Disease in a Taiwanese Population: A 10-year Follow-Up Study

**DOI:** 10.1186/s12872-023-03406-5

**Published:** 2023-07-28

**Authors:** Hsuan-Chih Tsai, Po-Sheng Hsu, Lung-Fa Pan, Chia-Lien Hung, Deng-Ho Yang, Kuang-Chen Hung, Chun-Cheng Liao

**Affiliations:** 1 Department of Family Medicine, Taichung Armed Forces General Hospital, No. 348, Sec. 2, Zhongshan Rd., Taiping Dist, Taichung, 41148 Taiwan; 2 Department of Occupational Medicine, Taichung Veterans General Hospital, Taichung, 40705 Taiwan; 3 School of Medicine, National Defense Medical Center, Taipei, 11490 Taiwan; 4 Department of Medical Education and Research, Taichung Armed Forces General Hospital, Taichung, 41148 Taiwan; 5 Department of Medical Imaging and Radiological Science, Central Taiwan University of Science and Technology, Takun, Taichung, 40601 Taiwan; 6 Department of Cardiology, Taichung Armed Forces General Hospital, Taichung, 41148 Taiwan; 7 Department of Medical Laboratory Science and Biotechnology, Central Taiwan University of Science and Technology, Taichung, 40601 Taiwan; 8 Department of Surgery, Zuoying Branch of Kaohsiung Armed Forces General Hospital, Kaohsiung, 813204 Taiwan; 9 Department of Surgery, National Defense Medical Center, Taipei, 11490 Taiwan; 10 Department of Surgery, Taichung Armed Forces General Hospital, Taichung, 41148 Taiwan; 11 Central Taiwan University of Science and Technology, Taichung, 40601 Taiwan; 12 National Chin-Yi University, Taichung, 411030 Taiwan

**Keywords:** Hyperglycemia, Mortality, Heart disease, Pre-DM

## Abstract

**Background:**

We evaluated hyperglycemia-associated mortality in the Taiwanese population by conducting a 10-year retrospective cohort study. Methods: From 2007 to 2017, all participants, regardless of their age or underlying diseases, were identified at a Health Screening Center in Taiwan. Overall, 114,534 participants were included in the analysis. They were classified into three subgroups according to glycemia and smoking status by combining survival for data analysis. Results: The mean follow-up time, age, and body mass index (BMI) were 8.14 ± 2.22 years, 40.95 ± 12.14 years, and 23.24 ± 3.65 kg/m^2^, respectively. The cumulative death rate increased from 0.9% in the normal fasting blood glucose(FBG) subgroup to approximately 6% in the diabetes FBG subgroup. After adjusting for age, gender, BMI, high-density lipoprotein, triglycerides, waist circumference(WC), and smoking status, the hazard ratio (HR) for all-cause, cancer, and heart disease mortality in the diabetes mellitus(DM) subgroup was 1.560, 1.381, and 1.828, respectively.HR was 0.989 in all-cause, 0.940 in cancer, and 1.326 in heart disease in the pre-DM subgroup.

**Conclusion:**

Being tested for pre-DM is related to a higher risk of death from heart disease in the Taiwanese population at baseline. Therefore, cardiovascular risk must be actively measured among diabetes patients every visit.

## Introduction

Several global challenges are related to hyperglycemia, and the most important challenge is diabetes mellitus (DM). The International diabetes foundation (IDF) released the 10th edition (latest edition) in 2021, confirming that diabetes is one of the fastest growing issues in the 21st century, with an estimate of 537 million adults with type 2 DM. IDF predicted that the diabetes population will increase to 51% in 2045, and it indicated that globally, 783 million people will have diabetes in 2045. Approximately 541 million people were estimated to have impaired glucose tolerance in 2021. Moreover, IDF had estimated that more than 6.7 million people aged 20–79 would die due to diabetes-related causes in 2021. Furthermore, [1, 2]. Lifestyles have changed in the modern economy; people are becoming physically sedentary and eating more carbohydrates. The healthcare system, especially in Taiwan, is threatened by the increasing prevalence of hyperglycemic population. Most healthcare-related costs are covered by national health insurance. Previous studies reported that insulin resistance was the main reason for the increasing number of patients with DM in Asia [3].

DM causes two types of vascular complications: microvascular and macrovascular. Macrovascular complications include coronary artery disease, ischemic cerebrovascular disease, and peripheral arterial disease (PAD), whereas the microvascular complication includes retinopathy. DM worsens and accelerates the course of atherosclerotic cardiovascular disease (CVD). Moreover, it is associated with an increased predisposition to the formation of modified lipoproteins, which are more atherogenic. In addition, oxidatively modified LDL particles are phagocytized by macrophages, leading to the formation of foam cells. S [4–6]. Furthermore, the increased risk of atherosclerosis in the population with DM characteristically results in several consequences: (1) A higher risk of stent restenosis following endovascular treatments, such as percutaneous coronary intervention and percutaneous transluminal angioplasty; (2) A higher severity of peripheral arterial disease in patients with peripheral arterial disease coexisting with diabetes; and (3) A higher amputation rate in patients with peripheral arterial disease coexisting with diabetes [7].

Similarly, hyperglycemia is associated with cognitive morbidity and mortality [8–10]. Globally, patients with DM have a higher mortality rate than those without diabetes [11]. Moreover, an Asian study indicated that patients with DM are more susceptible to cancer, with a prevalence rate of 8–18% [12, 13].

However, among patients with DM, hyperglycemia is rarely listed as the first cause on the death certificate. .

. We introduce a novel article analyzing the hyperglycemic risk of Pre-DM and DM among all-cause mortality, heart disease mortality and cancer mortality in middle-aged Asian populations.

It was well-known in the literature that DM increases all-cause mortality. However, the impact of DM and pre-DM on difference cause of mortality is uncertain.

Therefore, this study aimed to evaluate whether DM and pre-DM increased different cause of mortality in the Taiwanese population after 10 years follow up.

## Methods

### Study population

This retrospective cohort study retrieved data from a large private health examination institute (MJ Health Screening Center) in Taiwan from 2007 to 2017. The institute offers paid health examinations in large districts in Taiwan with a general payment of approximately USD 200–730. The demographics of the enrolled patients were assumed to be similar to those of the general Taiwanese population [14].

**Fig. 1:**
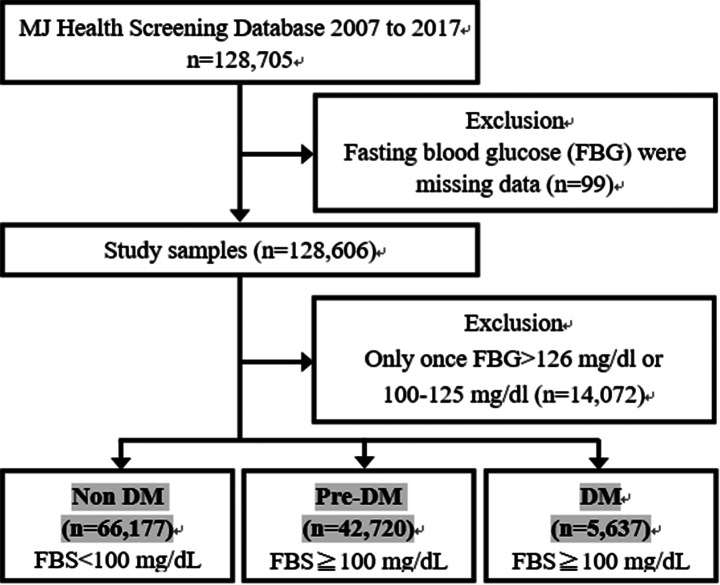
Flowchart of participants enrolment

In total, 114,534 participants, including 56,885 males and 57,649 females, had undergone the annual health examination at the MJ Health Screening Center, and they were followed up for at least 8.14 ± 2.22 years. We retrived twice blood glucose data from two healthchecks at different times. (Fig. 1)The mortality rate was verified in 2021 using the Death Registration from the ministry of Health and Welfare in Taiwan. The cause of death was defined in the tenth revision of the *International Classification of Disease* (ICD)-10, including heart diseases (ICD-10-CM code I01-I02.0, I05-I09, I20-I25, I27, I30-I52) and cancers (C00-C97). Survival years were estimated from 1996 to 2021.We retreived death records from 2007 to 2017.

Data used in this research were authorized by the MJ Health Research Foundation (Authorization Code: MJHRF2019016A) and were received from the same foundation. This study was approved by the Tri-service General Hospital Institutional Review Board (number: A202005160), and it was conducted in accordance with the principles stated in the Declaration of Helsinki. The Tri-service General Hospital is under the supervision of the School of medicine, National Defense Medical Center. Any interpretations or conclusions presented in this paper do not represent the views of the MJ Health Research Foundation. All participants in the research provided written informed consent before the health examination to authorize the data analysis. Personal identification data were removed by the MJ Health Research Foundation; hence, the participants remained anonymous during the entire research process. Details of the study population and data collection are described and reported elsewhere.

*2.2. Definition of Baseline Parameters*:

Clinical data and baseline parameters, including BMI, blood pressure, WC, high-density lipoprotein (HDL), triglycerides (TG), and fasting blood glucose (FBG), were collected. The Homogeneous Direct method (TOSHIBA C8000) was used for HDL cholesterol, the GPO-POD-ESPT method (TOSHIBA C8000) was used for TG level, and the HK.G-6-PD.NADP method (TOSHIBA C8000) was used for FBG level. Waist measurements of the upper hip bone and the bottom of the ribs, with no clothing interfering with the measurement were taken at the center. Smoking status were defined as follow: non-smoker, second hand smoker, smoker and quit smoking (individuals ever had cigarettes exposure) .

### Definition of DM and hyperglycemia

In 2022, the American Diabetes Association (ADA) defined a FBG level of > 100 mg/dl as hyperglycemia. Moreover, FBG levels of 100–125 mg/dl (5.6–6.9 mmol/dl) could be used for the diagnosis of prediabetes, whereas patients with twice the defined FBG levels, i.e., those with FBG of > 126 mg/dl (7.0 mmol/L) were defined diabetes mellitus. Fasting was defined as no caloric intake for ≥ 8 h [15].

### Subgroup analysis

We classified the patients into the following three subgroups according to their FBG status: diabetes mellitus (DM) subgroup: In twice separately tests, FBG values > 126 mg/dl; pre-diabetes mellitus (pre-DM) subgroup: FBG 100–125 mg/dl, and normal: FBG values < 100 mg/dl.

### Statistical analysis

The patients were examined from 2007 to 2017. The primary endpoint was the data of the cause of death, classified into all-cause mortality, mortality due to cancer, and mortality due to heart disease, associated with the death registry of the Ministry of Health and Welfare of Taiwan. An independent t-test was performed to compare follow-up time, age, and BMI between the participants with and without survival. We constructed normal and abnormal categories of WC, TG, and HDL levels by combining survival for data analysis. Furthermore, we constructed three subgroups of FBG level and smoking status by combining survival for data analysis. The Pearson chi-square test was used for dichotomous variables. Kaplan–Meier (KM) curve was used to analyze the cumulative survival of each category (all-cause mortality, mortality due to cancer, and mortality due to heart diseases). Crude hazard ratio (HR) with 95% confidence intervals (95% CI) was estimated using Cox regression models to assess the association between FBG levels and the risk of mortality. Finally, cox proportional hazards regression models were used to estimate the HR and 95% CI for mortality risk between the normal FBG, pre-DM, and DM subgroups after adjusting for gender, age, BMI, FBG, smoking status, HDL, TG, and WC. A forest plot was used to compare the risk between all-cause mortality, mortality due to heart disease,, and mortality due to cancer. The SPSS 22.0 (IBM, Armonk, NY, USA) was used for statistical analysis. The p-value of < 0.05 indicated statistical significance.

## Results

### Characteristics of the participants

Overall, 114,534 participants were included in the analysis. Basic patient characteristics and the distribution of demographics by FBG status are summarized in Tables 1 and 2, respectively. The means of follow-up time, age, and BMI were 8.14 ± 2.22 years, 40.95 ± 12.14 years, and 23.24 ± 3.65 kg/m^2^, respectively. During the follow-up, 1579 (1.4%) cases died. Among the participants, significant differences (p < 0.001) were observed in the cumulative incidences of mortality between BMI, WC, FBS, TG, HDL, gender, and smoking status. The cumulative incidence of mortality increased from 0.9% in the normal FBG group participants to 6.0% in the DM group participants (p < 0.001).

In Table 2, people with diabetes are older and have an increased BMI with gradual increase in value from normal-pre-diabetes-diabetes category. Inversion of the difference between normal and abnormal proportions observed in WC, TG and HDL. In addition, same trend is noted between quitted smoker and second-hand smoker.

**Table 1:** Distribution of the demographics

	Overall		Event	
		Living (n = 112,955)	Dead (n = 1,579)	P
Follow-up time, years	8.14 ± 2.22	8.16 ± 2.21	6.86 ± 2.41	< 0.001***
Age, years	40.95 ± 12.14	40.67 ± 11.87	59.79 ± 14.77	< 0.001***
BMI, kg/m^2^	23.24 ± 3.65	23.23 ± 3.64	24.09 ± 3.74	< 0.001***
Gender				< 0.001***
Male	56,885(49.7)	55,934(98.3)	951(1.7)	
Female	57,649(50.3)	57,021(98.9)	628(1.1)	
WC, cm	77.28 ± 10.32			< 0.001***
Normal		93,001(98.9)	1,062(1.1)	
Abnormal		19,464(97.4)	516(2.6)	
FBG, mg/dL	100.24 ± 18.96			< 0.001***
Normal		69,851(99.1)	608(0.9)	
Pre-DM		38,896(98.2)	724(1.8)	
DM		4,165(94.4)	247(5.6)	
TG, mg/dL	111.56 ± 92.11			< 0.001***
Normal		91,307(98.8)	1,141(1.2)	
Abnormal		21,639(98.0)	438(2.0)	
HDL, mg/dL	56.91 ± 14.91			< 0.001***
Normal		90,284(98.7)	1,186(1.3)	
Abnormal		18,093(98.1)	351(1.9)	
Smoking status				< 0.001***
Non-smoker	78,449(71.7)	77,513(98.8)	936(1.2)	
Second-hand smoker	4798(4.4)	4,762(99.2)	36(0.8)	
Smoker, Quit smoking	26,194(23.9)	25,688(98.1)	506(1.9)	

BMI: body mass index (kg/m^2^); WC: waist circumference (cm); FBS: fasting blood glucose (mg/dl); TG: triglyceride (mg/dl); HDL: high-density lipoprotein cholesterol (mg/dl). Smoking status: Second hand smoker, Smoker, quit smoking means current smoker or have been quitted smoking

Comparisons between were performed using independent t-test

*:p < 0.05; **:p < 0.01; ***:p < 0.001

**Table 2:** Distribution of the demographic by FBG status

					FBG status						
	Overall		Normal	Pre-DM		DM	P	Post Hoc			
Age, years	40.86 ± 12.08		38.00 ± 10.83	44.62 ± 12.39		53.68 ± 11.96	< 0.001***	Normal < Pre-DM Normal < DM Pre-DM < DM			
Follow-up time, years	8.22 ± 2.17		8.13 ± 2.20	8.15 ± 2.24		8.30 ± 2.15	< 0.001***	Normal < DM Pre-DM < DM			
Event							< 0.001***				
Living	112,955(98.6)		65,612(58.1)	41,994(37.2)		5349(4.7)					
Dead	1,579(1.4)		565(35.8)	726(46.0)		288(18.2)					
BMI, kg/m^2^	23.17 ± 3.62		22.33 ± 3.36	24.47 ± 3.54		26.29 ± 4.01	< 0.001***	Normal < Pre-DM Normal < DM Pre-DM < DM			
Gender							< 0.001***				
Male	56,885 (49.7)		25,446(44.7)	27,914(49.1)		3,525(6.2)					
Female	57,649 (50.3)		40,731(70.7)	14,806(25.7)		2,112(3.7)					
WC							< 0.001***				
Normal	94,063 (82.5)		59,862(63.6)	31,505(33.5)		2,696(2.9)					
Abnormal	19,980 (17.5)		5,975(29.9)	11,076(55.4)		2,929(14.7)					
TG							< 0.001***				
Normal	92,448 (80.7)		58,884(63.7)	30,667(33.2)		2,897(3.1)					
Abnormal	22,077 (19.3)		7,287(33.0)	12,050(54.6)		2,740(12.4)					
HDL							< 0.001***				
Normal	91,470 (83.2)		55,223(60.4)	32,729(35.8)		3,518(3.8)					
Abnormal	18,444 (16.8)		8,189(44.4)	8,440(45.8)		1,815(9.8)					
Smoking status							< 0.001***				
Non-smoker	78,449 (71.7)		47,794(60.9)	27,232(34.7)		3,423(4.4)					
Second-hand smoker	4798 (4.4)		2,989(62.3)	1,616(33.7)		193(4.0)					
Smoker, Quit smoking	26,194 (23.9)		12,599(57.9)	11,911(45.5)		1,684(6.4)					

BMI: body mass index (kg/m^2^); WC: waist circumference (cm); Abnormal WC was > 90 cm in men; WC > 80 cm in women.FBS: fasting blood sugar (mg/dl); TG: triglyceride (mg/dl); referance less than 150 milligrams per deciliter (mg/dL) HDL: high-density lipoprotein (mg/dl). Reference Range · Male: >45 mg/dL or > 0.75 mmol/L (SI units) · Female: >55 mg/dL or > 0.91 mmol/L (SI units)

Age, follow-up time, and BMI were compared between the groups classified by the FBG status by performing an analysis of variance, followed by Scheffe’s post-test. Sex, WC, TG, HDL, and smoking status were compared using a chi-square test

*:p < 0.05; **:p < 0.01; ***:p < 0.001

### Cox regression analysis of mortality risk in patients with and without DM

Table 3 shows the association between DM and the risk of mortality. Compared with the normal FBG subgroup, significantly increased risk of all-cause mortality was noted in the pre-DM (HR: 1.982, 95% CI: 1.795–2.187) and DM subgroups (HR: 6.209, 95% CI: 5.427–7.104). Similar results were observed when we used a separate analysis for mortality risk assessment due to cancer and heart disease.

**Table 3:** Cox regression analysis of mortality risk in patients with and without DM

	Mortality of all cause			Mortality of cancer			Mortality of Heart Disease		
	Crude HR	95%CI		Crude HR	95%CI		Crude HR	95%CI	
		lower	upper		lower	upper		lower	upper
FBG status									
Normal	ref.			ref.			ref.		
Pre-DM	1.982***	1.795	2.187	1.785***	1.551	2.054	2.924***	2.103	4.066
DM	6.209***	5.427	7.104	4.986***	4.061	6.122	9.222***	5.973	14.238

FBG: fasting blood glucose; DM: diabetes mellitus; HR: hazard ratio

*:p < 0.05; **:p < 0.01; ***:p < 0.001

**Fig. 2:**
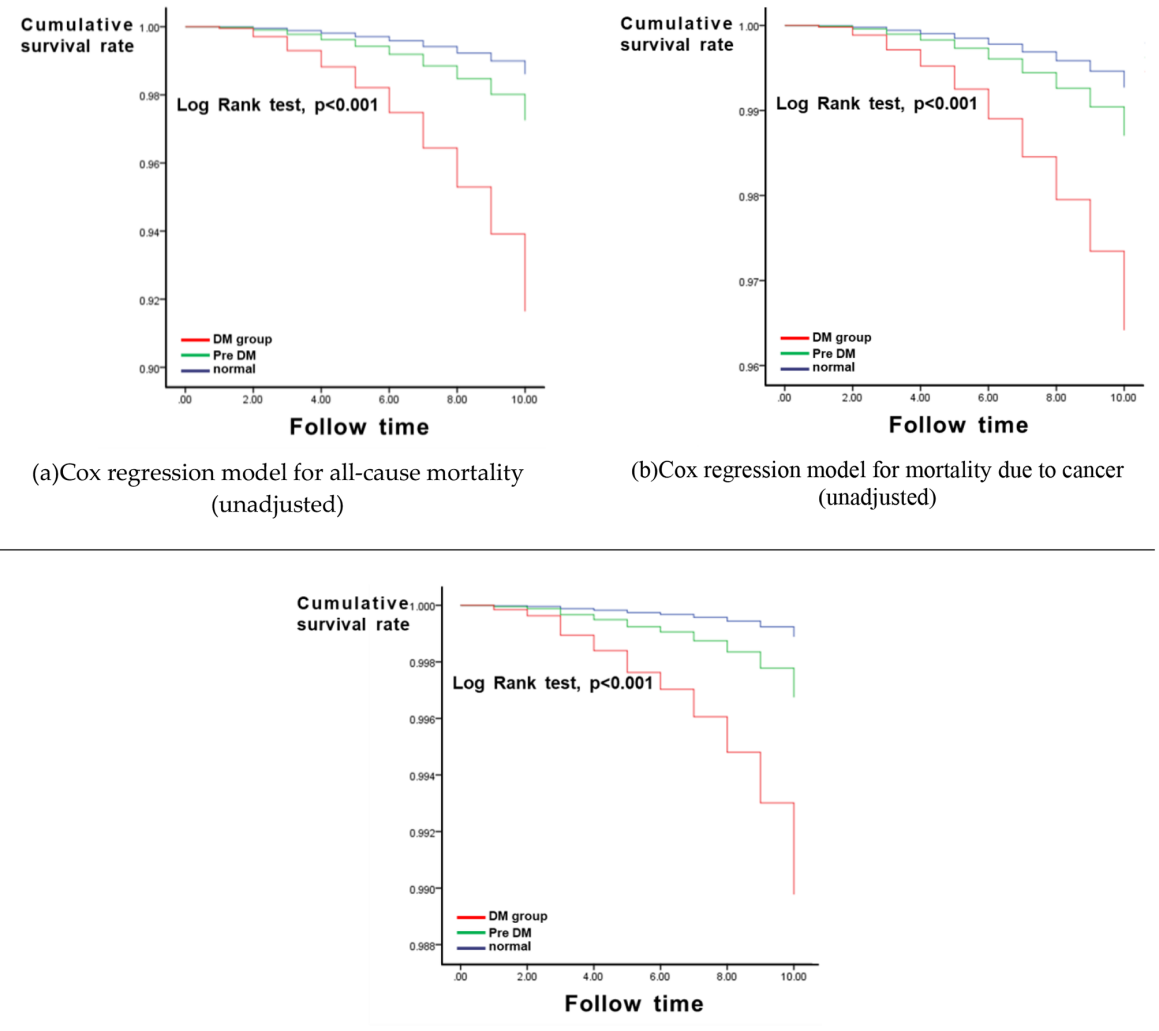
Cox regression model for mortality (unadjusted) among (**a**) all-cause, (**b**) cancer, (**c**) and heart disease

Moreover, we used cox proportional-hazards model with Kaplan–Meier (KM) curve to analyze the cumulative survival of each category (all-cause mortality, mortality due to cancer, and mortality due to heart diseases); these are presented in Fig. 2. Regardless of whether we compared the cumulative survival of the all-cause mortality category, cancer, or heart disease category, after approximately 10 years of follow-up, we were able to see a significant decrease in cumulative survival in the DM group versus the pre-DM and normal subgroups.

### Pooled estimated of adjusted HRs for mortality cause according to categories of FBG subgroups

**Fig. 3:**
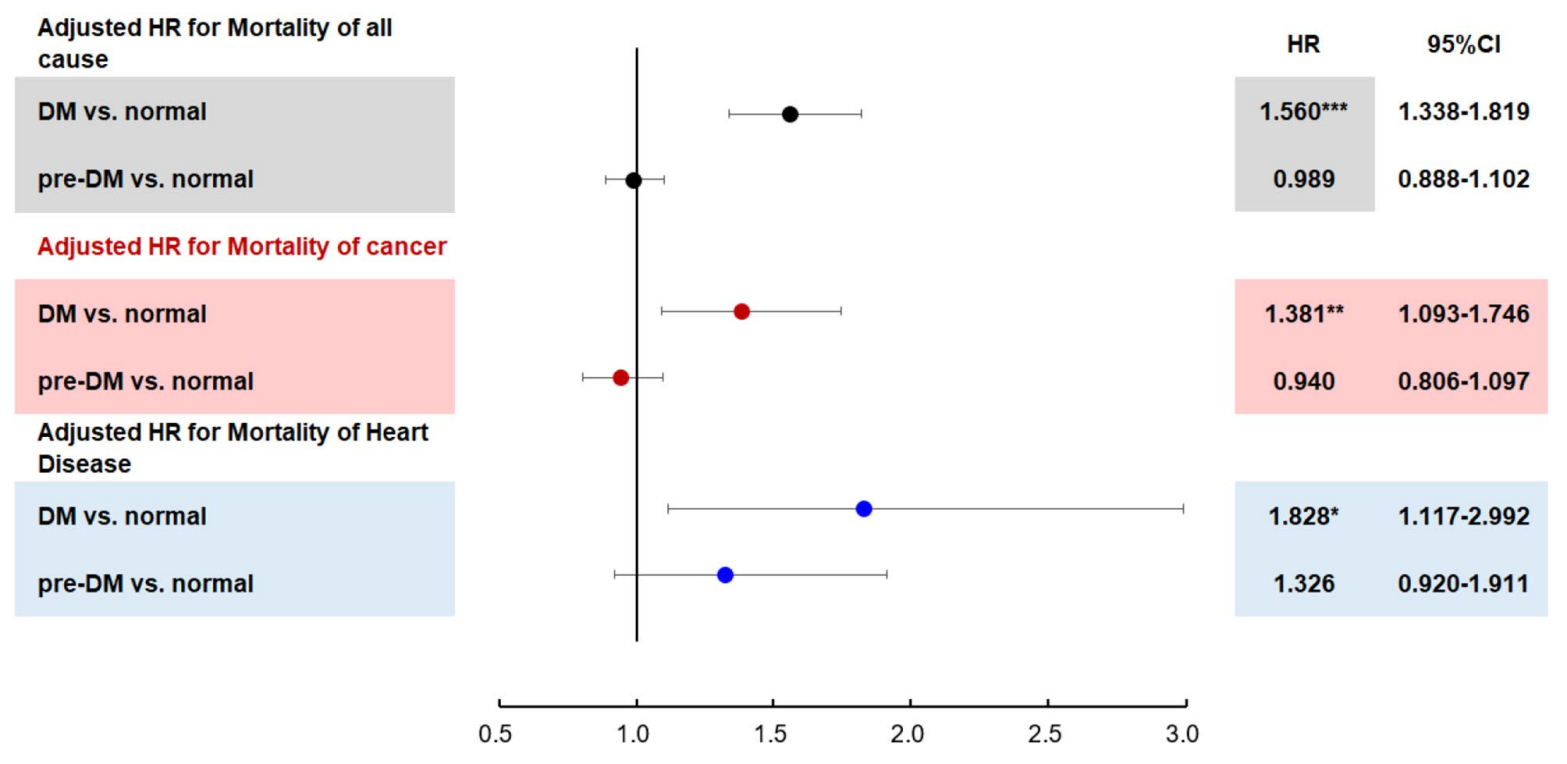
Forrest plot of hazard ratio of mortality by Cox regression. HRs were adjusted for age, gender, BMI, HDL, TG, WC, and smoking status *:p < 0.05; **:p < 0.01; ***:p < 0.001

Figure 3 displays another pooled estimate of adjusted HRs for all-cause mortality, heart disease mortality, and cancer mortality according to the categories of FBG. Compared to the normal FBG subgroup, the HR for all-cause mortality in DM subgroup was 1.560 (95% CI: 1.338–1.819, p < 0.001). A significantly higher risk of heart disease-related mortality was observed in participants of the DM subgroup (HR: 1.828, 95% CI: 1.117–2.992, p < 0.001). When we focused on the HRs for cancer-related mortality, the DM subgroup had significantly higher rates than the normal FBG subgroup (HR: 1.381, 95% CI: 1.093–1.746, p < 0.05).

We can see the data on Fig. 3,Compared to the normal FBG subgroup, the HR for all-cause mortality in pre-DM subgroup was 0.989 (95% CI: 0.888–1.102, p > 0.05), there was no significantly increase. Compared to the normal FBG subgroup, the HR for heart disease-related mortality in pre-DM subgroup was 1.326 (95% CI: 0.920–1.911, p > 0.05), there was no significantly increase. When we focused on the HRs for cancer-related mortality, the pre-DM subgroup also didn’t have significantly higher rates than the normal FBG subgroup 0.940 (95% CI: 0.806–1.097, p > 0.05).

The HRs for mortality causes between the pre-DM and normal FBG subgroups did not significantly increase after adjusting for age, gender, BMI, HDL, TG, WC, and smoking status.

## Discussion

Current study of a general Taiwanese population showed that fasting blood glucose levels within the pre-diabetes range (100–125 mg/dl) do not significantly increase the risk of all-cause mortality, cancer, or heart disease. However, once pre-diabetes progresses to diabetes (FBG > 125 mg/dl), there is a significant increase in the risk of all-cause mortality, cancer, and heart disease. Therefore, it is important to prevent the progression of pre-diabetes to diabetes through lifestyle changes such as healthy diet and regular exercise.

There is one meta-analysis conducted by China researchers including 31,662 article, 129 studies. 75% of studies are in Europe and Asia. The results demonstrated that having prediabetes will increase the risk of heart disease (relative risk 1.09; 1.04–1.14 95% CI). In comparison, prediabetes increases the risk of all-cause mortality with similar results to heart disease (RR 1.07, 1.03–1.12 95% CI). Xiaoyan Cai et al. revealed all-cause mortality had relative risk of 1.03 in pre-DM group. In the subgroup analysis in above Chinese study, participant’s aged < 60 had relative risk of 1.16 [16]. In addition, Chinese researchers found similar HR with 0.89 in all-cause mortality after adjusted for age, sex, BMI, cigarette smoking, blood pressure and HDL cholesterol, LDL cholesterol and triglycerides in same population as this study. Though, the mean age was 57.7 ± 8.9 [17]. In our study, the overall average of participant’s was 40.86 ± 12.08. Scarcely the relative risk between all-cause mortality and heart disease. In addition, another meta-analysis conducted by European researchers depict being pre-diabetes had a HR 1.27 in CV mortality (1.02–1.58 95% CI) [18].

When humans is in postparidal status, our islet β cells in pancrease produce insulin into blood system for blood glucose control. The bondary of insulin and insulin receptors in cell membranes facilitate glucose uptake by the cell. As a result, leading to the decreased of blood glucose. Deteroirtion of the insulin production in islet β cells or dysfunction of the regulation between glucose transporter translocation in cell membrane, or both, lead to hyperglycemia. Anminal studys in late twentieth century revealed an increased in inflammation mediators ,for example, the tumor necrosis factor( TNF)- α, interleukin(IL)-6, C-reative protein among obese mice [19, 20]. Insulin receptor substrate(IRS)-1 and − 2 tyrosine phosphorylated after the binding of insulin and insulin receptor regulated the normal glucoe metabolism. Insulin resistance was casused by Serine phosphorylation of IRS substrates by jun N-terminal kinase (JNK1), and inhibitor of NF-κB-kinase β (IKKβ, an NF-κB-activating kinase) [21] Decreased insulin secretion and insulin resistance leads to hyperglycemia.

Hyperglycemia may be detected using fasting blood glucose levels, oral glucose tolerance test, and HbA1c levels. These three methods measure different physiological processes. A1c has stablity and measurability in randaom versus fasting blood glucose. However, A1c could be affected by genetically variation of hemoglobin structure [22].A Dutch study by vant Riet et al. found that the correlation coefficient between glucose tolerance test and HbA1c levels differs between known diabetes, newly diagnosed diabetes, and impaired glucose tolerance [23]. The information about the measurement of glycemia remains unclear [24]. Additionally, a study from India reported considerable disagreement regarding the use of the FBG criterion (FBG > 126 mg/dl) or HbA1c ≥ 6.5% to determine DM [25] The HbA1c and glucose correlation coefficients were either insignificant or uncorrelated [23] The result of Outcome Reduction with Initial Glargine Intervention (ORIGIN) trial, account > 12,500 people, documented clear graded relationship between FBG ≥ 100 (5.6mmol/L) and A1c levels. And is unaffected by geography or ethnic group [26].Among our study population, most cases were of impaired glucose tolerance (FBG: 100.24 ± 18.96; Table 1).Thailand researchers stuided correlation between HbA1c and FBG in 3 hemoglobulin group(normal, hbE and high hbA_2_), came to conclusion with a positive association between estimated average glucose and FBG. Estimated averge glucose was calculated through equation from HbA1c [27].

German study investigating the impact of hyperglycemia on remodeling of the heart by cardiac MR imaging, which include 390 individuals (39%) was prediabetes and 49 (4.9%) were found to have unknown type 2 diabetes. A positive association was established between hyperglycemia and hypertrophy of the left ventricle. Net effect of hyperglycemia resulted in a concentric of left ventricle, which was unconstrained of other confounders, such as hypertension. To summarized, concentric remodeling of left ventrile result in arterial stiffness, smaller left ventricle size and higher thickness of left ventricle [28]An Asian study revealed that the fasting hyperglycemia was a risk factor of atrial fibrillation in patients with acute myocardial infarction [29]. During Coronavirus Disease 2019 (COVID-19) pandemic, a study found an increased deathrate in patients with new-onset hyperglycemia without diabetes, than those having pre-existing diabetes (41.7% vs. 14.8%, respectively; <0.001).

Saudi Arabia researchers conducted 679 recruited anticipants, with approximately the same age with this study. 32 ± 11.8,40 ± 12.14, respectively [30]. BMI and TG showed correlation with pre-diabetes, but not in the HDL variance and smoking status. Another middle east study investigates a city with approximately 3.5 million populations, with mean age 35.7 ± 15.44 years, reported BMI, WC, and dyslipidemia are correlated with DM. However, dyslipidemia merely reach statistic significant (P = 0.004 in men and 0.064 in women) in pre-diabetes. Neither the smoking status nor WC [31]the differences in correlation between hyperglycemia and dyslipidemia cross studies might be attributed to the different methods used for defining prediabetes, as well as the difference in age between the populations studied.

The current study findings provide epidemiological evidence indicating that in the Taiwanese population, experiencing Pre-DM (FBG 100-125 mg/dl) is associated with increased mortality due to heart disease. Using FBG data in large number of populations, with the Death Registration from ministry of Health and Welfare in Taiwan, and a retrospective follow-up of 10 years, we were able to examine and compare hyperglycemia with pre-DM and DM related mortality issues in Taiwan. The results of the current study form a cornerstone of highlightening the heart-relate mortality issue in pre-diabete group.

[32]

The HRs for all-cause mortality in the DM subgroup, regardless of age and sex (Table 2; Fig. 3), demonstrated a similar results to that of the Sweden cohort [33]. The increased mortality rate among DM population have been reported since 1985 [34]. The higher risk of mortality among DM people is brought on by the cardiovascular consequences of DM. The Multiple Risk Factor Intervention Trail Group trials showed how diabetes affects cardiovascular system of males between the ages of 35 and 57. Using the fasting blood glucose as a subgroup, Eberly et al. demonstrated that a fasting glucose level greater than 126 mg/dl predicts an increased risk of mortality [35, 36]. Another recent study by Mansor et al., using the US National Health and Nutrition Examination Survey data, revealed that males aged 30–84 years with diabetes had a higher mortality rate than females from 1999 to 2010 [37].

The cumulative survival of the pre-DM and DM subgroups significantly decreased after approximately 10 years in the cox proportional-hazards model using the Kaplan–Meier (KM) curve of all-cause mortality, heart disease, and cancer mortality (Fig. 2). Fortunately, the advancement in technology led to a decrease in death rates due to vascular disease among individuals with [38–41] and without diabetes [39, 42].A similar trend was also reported in Asian countries [43].

In summary, the current study has three main strengths. First, to the best of our knowledge, this is the first study with youngest age of participants to be conducted in Taiwan that shows how hyperglycemia(DM or pre-DM) affects mortalities. Second, the profound effect of deteriorated glucose metabolism started from heart disease. Mortality from heart disease had higher HR than all-cause mortality and cancer in pre-diabetes populations. The influence of having a DM diagnosis increase the all-cause mortality was well-established in the NHID database [40–43]. The presence of DM appears to be a risk factor for all-cause mortality and mortality due to cancer and heart disease worldwide [14, 42]. Third, the population size enrolled in our study is sufficient to represent the general Taiwanese population. Forth, The ethnic groups we studied represent the most important productive forces in a country, our study demonstrate how pre-DM or DM impact those young adults from the begining.

This study also had some limitations.

First, the lack of sufficient HbA1c data in our database stems from the fact that HbA1c is not a routine test among the MJ Health Screening Center .Hence, we chose FBG instead of HbA1c for subgroup analysis. Additionally, FBG is more immediate and accessible in day-to-day practice.

Second, we were unable to discover the detailed competing cause of death, socio-economic factors the drug record, amount of cigarette exposure and detailed occupational data of each individual in this study. The MJ database was compulsive with adult health examination, which is paid by Taiwan government and self-paid physical health examination. Third, the cause of mortality, especially in heart disease and cancer, we can’t precisely classify heart disease and type of cancer.

## Conclusions

The current study offers epidemiological evidence indicating that being tested as diabetes or pre-DM, is linked to a higher mortality due to heart disease than all-cause and cancer in the Taiwanese population. How to early implement heart prevention in those pre-diabetes population is a public issue for government in Taiwan.Moreover, more precautions should be adopted for pre-DM populations (FBG 100–125 mg/dl) because the all-cause mortality risk will be increased approximately 50% when they progress to be diagnosed with DM.

## Data Availability

Data are available from the MJ Health Research Foundation. Due to the legal restrictions imposed by the government of Taiwan in relation to the “Personal Information Protection Act,” data cannot be made publicly available.

## Abbreviations

BMI: Body mass undex
FBG: Fasting blood glucose
WC: Waist cirvumference
HR: Hazard ratio
DM: Diabetes mellitus
IDF: International diabetes foundation
PAD: Peripheral arterial disease
CVD: Cardiovascular disease
ICD: International Classification of Disease
HDL: High-density lipoprotein
TG: Triglycerides
ADA: American Diabetes Association
KM: Kaplan–Meier
HR: Crude hazard ratio
